# Supplementary material for: COVID-19 IgG seropositivity and its determinants in occupational groups of varying infection risks in two Andean cities of Ecuador before mass vaccination
Source: PLoS One. 2024 Aug 29;19(8):e0309466. doi: 10.1371/journal.pone.0309466 (PMC11361580; doi:10.1371/journal.pone.0309466)
Supplement: S1 File — (DOCX) [file pone.0309466.s001.docx]

**Supporting Information**

**S1 File**

**Cuestionario ISARIC-MÓDULO 1**

1. Código del participante
2. Código del entrevistador
3. Cohorte de investigación
   1. Riesgo Alto
   2. Riesgo Moderado
   3. Riesgo Bajo
   4. Riesgo Indeterminado
4. Datos Demográficos
   1. Lugar de encuesta
   2. Número de Teléfono
   3. Fecha de inicio de participación/Fecha de la primera evaluación de la COVID-19
      1. yyyy-mm-dd
   4. Grupo étnico
      1. Mestizo
      2. Montubio
      3. Indígena
      4. Afrodescendiente
      5. Blanco
      6. Otro
      7. No sabe
5. ¿Trabaja como personal sanitario?
   1. Sí
   2. No
   3. No sabe
6. ¿Trabaja en un laboratorio de microbiología?
   1. Sí
   2. No
   3. No sabe
7. Sexo biológico
   1. Varón
   2. Mujer
   3. No especificado/Se desconoce
8. Edad (años)
9. ¿Embarazo?
   1. Sí
   2. No
   3. No sabe
10. Valoración de las semanas de gestación
11. Historial de fiebre
    1. Sí
    2. No
    3. No sabe
12. Tos
    1. Sí - sin esputo
    2. Sí - con esputo
    3. Sí - con hemoptisis
    4. No
    5. No sabe
13. Dolor de Garganta
    1. Sí
    2. No
    3. No sabe
14. Goteo nasal (rinorrea)
    1. Sí
    2. No
    3. No sabe
15. Sibilancias
    1. Sí
    2. No
    3. No sabe
16. Falta de aliento
    1. Sí
    2. No
    3. No sabe
17. Tiraje
    1. Sí
    2. No
    3. No sabe
18. Dolor torácico
    1. Sí
    2. No
    3. No sabe
19. Conjuntivitis
    1. Sí
    2. No
    3. No sabe
20. Linfoadenopatías
    1. Sí
    2. No
    3. No sabe
21. Cefalea
    1. Sí
    2. No
    3. No sabe
22. Pérdida del olfato (anosmia)
    1. Sí
    2. No
    3. No sabe
23. Pérdida del gusto (ageusia)
    1. Sí
    2. No
    3. No sabe
24. Convulsiones
    1. Sí
    2. No
    3. No sabe
25. Fatiga/malestar
    1. Sí
    2. No
    3. No sabe
26. Anorexia
    1. Sí
    2. No
    3. No sabe
27. Estado de conciencia alterado/confusión
    1. Sí
    2. No
    3. No sabe
28. Dolor muscular (mialgia)
    1. Sí
    2. No
    3. No sabe
29. Dolor articular (atralgia)
    1. Sí
    2. No
    3. No sabe
30. Incapaz de andar
    1. Sí
    2. No
    3. No sabe
31. Dolor abdominal
    1. Sí
    2. No
    3. No sabe
32. Diarrea
    1. Sí
    2. No
    3. No sabe
33. Vómitos/náuseas
    1. Sí
    2. No
    3. No sabe
34. Erupción cutánea
    1. Sí
    2. No
    3. No sabe
35. Sangrado (hemorragia)
    1. Sí
    2. No
    3. No sabe
       1. If sí: Especificar sitio o sitios
36. Otros síntomas
    1. Sí
    2. No
    3. No sabe
       1. If yes, Especificar
37. ¿ Ha tenido Covid-19?
    1. Si
    2. No
38. ¿En qué fecha tuvo COVID-19?
    - 1. yyyy-mm-dd
39. ¿Cómo le diagnosticaron?
    1. Examen de laboratorio
    2. Examen de Imagen
    3. Por clínica, sin exámenes de laboratorio o imagen
    4. Usted pensó que tuvo Covid-19
40. ¿Cual tipo de imagen?
    1. TAC
    2. RX
    3. Otro
    4. Especifique
41. Si usted tuvo Covid-19
    1. Tuvo que ser hospitalizado en el área general para Covid-19
    2. Tuvo que ser hospitalizado en UCI para Covid-19
    3. Paso en casa
42. Durante el ingreso hospitalario el paciente recibió o fue ingresado en UCI o unidad de cuidados intermedios
    1. Sí
    2. No
    3. No sabe
43. Duración total del ingreso en días
    1. Si conoce
    2. No conoce
44. Indique la duración del ingreso en días
45. Fecha de ingreso a UCI
    1. Si conoce
    2. No conoce
46. Indique la fecha de ingreso a UCI
    1. yyyy-mm-dd
47. Fecha del alta de UCI
    1. Si conoce
    2. No conoce
48. Indique la fecha del alta de UCI
    1. No se dio el alta
    2. yyyy-mm-dd
49. Número de muestras que se tomó para el diagnóstico
    1. Ninguna
    2. 1
    3. 2
    4. 3
    5. 4
50. Fecha de recogida de la primera muestra para el diagnóstico
    1. yyyy-mm-dd
51. Tipo de muestra biológica
    1. Muestra nasal/NF
    2. Muestra nasal/NF + muestra faríngea comb
    3. Esputo
    4. Aspirado traqueal
    5. Heces/frotis rectal
    6. Muestra faríngea
    7. Lavado broncoalveolar
    8. Orina
    9. Sangre
    10. Otro
        1. Especificar
52. Método de prueba analítica
    1. PCR
    2. Cultivo
    3. Otro
       1. Especificar
53. Resultado
    1. Positivo
    2. Negativo
    3. No sabe
    4. Dudoso
54. Patógeno analizado/detectado
    1. SARS-CoV-2
    2. Otro
       1. Especifique
55. Fecha de recogida de la segunda muestra para el diagnóstico
    1. yyyy-mm-dd
56. Tipo de muestra biológica
    1. Muestra nasal/NF
    2. Muestra nasal/NF + muestra faríngea comb
    3. Esputo
    4. Aspirado traqueal
    5. Heces/frotis rectal
    6. Muestra faríngea
    7. Lavado broncoalveolar
    8. Orina
    9. Sangre
    10. Otro
        1. Especificar
57. Método de prueba analítica
    1. PCR
    2. Cultivo
    3. Otro
       1. Especificar
58. Resultado
    1. Positivo
    2. Negativo
    3. No sabe
    4. Dudoso
59. Patógeno analizado/detectado
    1. SARS-CoV-2
    2. Otro
       1. Especifique
60. Fecha de recogida de la tercera muestra para el diagnóstico
    1. yyyy-mm-dd
61. Tipo de muestra biológica
    1. Muestra nasal/NF
    2. Muestra nasal/NF + muestra faríngea comb
    3. Esputo
    4. Aspirado traqueal
    5. Heces/frotis rectal
    6. Muestra faríngea
    7. Lavado broncoalveolar
    8. Orina
    9. Sangre
    10. Otro
        1. Especificar
62. Método de prueba analítica
    1. PCR
    2. Cultivo
    3. Otro
       1. Especificar
63. Resultado
    1. Positivo
    2. Negativo
    3. No sabe
    4. Dudoso
64. Patógeno analizado/detectado
    1. SARS-CoV-2
    2. Otro
       1. Especifique
65. Fecha de recogida de la cuarta muestra para el diagnóstico
    1. yyyy-mm-dd
66. Tipo de muestra biológica
    1. Muestra nasal/NF
    2. Muestra nasal/NF + muestra faríngea comb
    3. Esputo
    4. Aspirado traqueal
    5. Heces/frotis rectal
    6. Muestra faríngea
    7. Lavado broncoalveolar
    8. Orina
    9. Sangre
    10. Otro
        1. Especificar
67. Método de prueba analítica
    1. PCR
    2. Cultivo
    3. Otro
       1. Especificar
68. Resultado
    1. Positivo
    2. Negativo
    3. No sabe
    4. Dudoso
69. Patógeno analizado/detectado
    1. SARS-CoV-2
    2. Otro
       1. Especifique
70. Desenlace
    1. Alta
    2. Ingreso
    3. Traslado a otro centro
    4. Fallecimiento
    5. Alta paliativa
    6. Finalización de aislamiento domiciliario
    7. No sabe
71. Fecha del desenlace
    1. Si conoce
    2. No conoce
72. Indique la fecha del desenlace
    1. yyyy-mm-dd
73. Capacidad de cuidar de sí mismo en comparación con su estado anterior a la enfermedad
    1. Igual que antes de la enfermedad
    2. Peor
    3. Mejor
    4. No sabe
74. Tratamiento tras el alta: Oxigenoterapia
    1. Si
    2. No
    3. No sabe
