# Supplementary material for: COVID-19 IgG seropositivity and its determinants in occupational groups of varying infection risks in two Andean cities of Ecuador before mass vaccination
Source: PLoS One. 2024 Aug 29;19(8):e0309466. doi: 10.1371/journal.pone.0309466 (PMC11361580; doi:10.1371/journal.pone.0309466)
Supplement: Attachment — Submitted filename: Response to Reviewers.docx [file pone.0309466.s002.docx]

“COVID-19 IgG Seropositivity and its determinants in occupational groups of varying infection risks in two Andean cities of Ecuador before mass vaccination”

Response to Reviewers

Dear Editor,

We would like to thank you for your consideration for publication of our manuscript in PLOS ONE. We would also like to thank you for the opportunity to reply to the reviewers’ comments, requests, and constructive criticisms.

We have addressed them all and we have also added the suggested, interesting references and altered the manuscript in light of their useful remarks. We now believe that the manuscript, the communication, and the scientific messages have greatly improved as result of all these reflections. Therefore, we very much hope that the manuscript is ready for publication. We have attached both clean and altered manuscripts with tracked changes in red, as requested.

The manuscript now fits PLOS ONE’s style requirements and we have included the questionnaire on inclusivity in global research. The acronym of CEDIA has been expanded in the acknowledgements section and reads: “Corporación Ecuatoriana para el Desarrollo de la Investigación y Academia” (CEDIA). In the same section, we have included the information for the Seroprevalence ECU Group as well as the lead author. Finally, regarding data availability, a simplified database can be accessed openly through: <https://bit.ly/4aa1Fdf>.

Dear Reviewers,

Thank you for your insightful comments and constructive suggestions. Below, please find a point-by-point response addressing your remarks.

**Reviewer #1**

**Comment 1:** I have several questions and also think that the authors should have focused more on the age (and sex) difference between the groups, regions and time of sampling in the discussion.

*Thank you for your insightful comment.*

*Serological data collection around the time of the pandemic had enormous difficulties. Matching by any variable would have added another layer of complexity which we thought was unrealistic at the time.*

*Therefore, our study has not been designed to be comparative. There are three very different occupational groups with a priory envisaged different characteristics and levels of exposure. We aimed at representing them and investigate their seropositivity. For instance, the university population was expected to be younger and healthier than other two, the hospital workers were envisaged to experience higher levels of exposure but also protection, whilst the latter group assumed to have had the average population levels of exposure. Therefore, we think that comparative seropositivity analysis would be misleading. Averaging across the three population would also encounter difficulties in defining the “population denominator”.*

**Comment 2:** I wonder why the group expected to have median exposure risk had significantly higher seropositivity than the other two groups. Why did the high-risk group have such a low seropositivity? Was it because they were mostly health care workers using personal protective equipment (PPE)? This could have been discussed further, se more below.

*Thank you for your insightful questions. We found the difference between the MR and HR groups very interesting as well and perhaps we were not discussing it enough. We have improved the discussion related to this beginning at line 279 that now reads: “In Ecuador, surveillance of SARS-CoV-2 infections was carried out by the Public Ministry of Health and reported in official websites and bulletins; from these, during the sampling period of our study (January to September, 2021), a peak in daily COVID-19 confirmed cases (2,085 new cases) was reported in the 27th of April, representing a 2.5-fold increase when compared to the 834 new cases reported in the 13th of January (the beginning of our sampling) [62, 63]. Therefore, our HR group was sampled during a period of low daily COVID-19 transmission and its sampling ended before the aforementioned peak”. Also, starting at line 294: “A study by Morales-Jadan et al., using RT-qPCR in four, mostly Andean, provinces of Ecuador (Bolivar, Chimborazo, Tungurahua, and Napo) observed an overall PCR positivity of 26.2% (95%CI, 23.6%-29%); however, when looking into the individual provinces, Tungurahua had the highest infection rate with 65.3% (95%CI, 58.7-71.4%) [58]. This finding is relevant for our study given that Ambato, where our MR group was recruited, is the capital city of Tungurahua; this might also explain the high seropositivity (74.6% [CI 95% 66.4-81.4]) we found in this group.” And finally, beginning at line 410: “Additionally, as mentioned before, a study done in June 2020, reported a high RT-qPCR positivity of 65.3% (95%CI, 58.7-71.4%) in Tungurahua, the province where Ambato is the capital city. On the other hand, the higher seropositivity in the MR group in comparison to our two other groups might also be due to the sampling period (i.e., it was the last one to be recruited), although there was some overlap with the LR group [1,33].”*

**Comment 3:** Does Ecuador have a registry of infection based on confirmed COVID-19? This would have been interesting to know. Also, if such a register exists, did it show a difference between different regions of Ecuador? (as this manuscript focuses on two cities).

*Yes, we have provided the information regarding the registry as well as the behavior of the epidemic during our sampling period in the discussion section starting at line 279 that reads: “In Ecuador, surveillance of SARS-CoV-2 infections was carried out by the Public Ministry of Health and reported in official websites and bulletins; from these, during the sampling period of our study (January to September, 2021), a peak in daily COVID-19 confirmed cases (2,085 new cases) was reported in the 27th of April, representing a 2.5-fold increase when compared to the 834 new cases reported in the 13th of January (the beginning of our sampling) [62, 63]. Therefore, our HR group was sampled during a period of low daily COVID-19 transmission and its sampling ended before the aforementioned peak. Afterwards, new daily cases stayed above 1,000 until the middle of July when they started to decrease steadily, reaching 219 new cases in the 27th of September (our last sampling date) [62, 63].”*

**Comment 4:** Abstract. Where is the number of infections and deaths reported in the first line of the abstract reported and when (reference)? These numbers are not repeated and referenced in the introduction. The numbers also seem a little high. According to worldometer on the 30th of August 2023, the numbers for South America was 69 million cases and 1.4 million deaths.

*Thank you for indicating this to us, we accept that more clarity is required. The referenced number was for Latin America, not South America. Latin America includes Mexico and other countries that are not part of South America. We have changed the numbers to reflect South America only, as requested and have provided the reference in the introduction starting in line 33 that now reads: “COVID-19, caused by the severe acute respiratory syndrome coronavirus 2 (SARS-CoV-2), emerged as a global pandemic estimated at 68.7 million infections and 1.35 million deaths in South America up to February 2024 [1].”*

**Comment 5:** In the objectives, the part about studying determinants potentially associated with seropositivity is not mentioned.

*Thank you for pointing this out. We have added this in the abstract starting at line 5 and now reads: “Objective: To estimate SARS-CoV-2 seropositivity and its determinants before vaccination in occupational groups of adults presumed to have different levels of exposure and associations with potential symptomatology.”*

**Comment 6:** In the conclusion, I would suggest using another word than “significant” to describe the proportions as this term if commonly used when discussing statistical significance.

*We agree with your assessment, we have changed the word to “Notable”, in line 22 of the abstract.*

**Comment 7:** Introduction. Lines 38-43 is one long sentence and the meaning is difficult to understand. I suggest that you rephrase.

*Thank you, we have rephrased it to improve clarity starting at line 42 and now reads: “Numerous studies have reported SARS-CoV-2 seropositivity in different populations worldwide prior to the implementation of vaccination campaigns [7–19]. However, there are relatively few studies from the South American region assessing pre-vaccination seropositivity in occupational risk groups [14, 20].”*

**Comment 8:** Lines 48-52, a bit detailed with all the different world regions, it is perhaps better to focus more on South/Latin America.

*We have focused more on Latin/South America, starting at line 50 and now reads: “Another meta-analysis of 88 studies from 34 countries, with sampling dates up to November 2020, estimated a pooled seropositivity of 8% (95% CI 6-10%) in the Americas that was highest in Colombia (29%, 95% CI 23-31%) [2]. Analysis of nine studies done in South America between April and September 2020 estimated a pooled seropositivity of 33.6% (95% CI 28.6-38.5%), [52] with a particularly high seropositivity of 73% observed in indigenous populations [10].”*

**Comment 9:** Line 58-59 states that it is limited information on seropositivity in occupational groups and associated factors. I guess you mean in Ecuador as line 59 and 56 have many references studying this.

*Yes, indeed, we meant in Ecuador, we have improved the clarity of this sentence starting at line 88 and that now reads: “There are limited published information on pre-vaccination COVID-19 seropositivity in occupational groups of varying infection risks and associated factors in Ecuador [12–19].”*

**Comment 10:** Materials and methods. I wonder if it is a “problem” that the participants came from different regions of Ecuador? What is known about the spread of the pandemic between different regions and time points?

*Thank you for your comment. Our objective was to assess different occupational groups and we wanted to include a group composed mainly of merchants/farmers, that was found in Huachi Grande, Ambato. A similar group could not have been possible to find in Quito that, as the capital of the country, has a more heterogenous population. Regarding the spread of the pandemic, within and between regions, there is limited published data in Ecuador. We have included more information of the community transmission and reported infection information of Ecuador during our sampling periods in the discussion starting at line 277 and reading: “It's important to interpret our data in the context of community transmission and SARS-CoV-2 attack rates in order to understand why we obtained high seropositivity amongst all groups, and in particular amongst the MR group. In Ecuador, surveillance of SARS-CoV-2 infections was carried out by the Public Ministry of Health and reported in official websites and bulletins; from these, during the sampling period of our study (January to September, 2021), a peak in daily COVID-19 confirmed cases (2,085 new cases) was reported in the 27th of April, representing a 2.5-fold increase when compared to the 834 new cases reported in the 13th of January (the beginning of our sampling) [62, 63]. Therefore, our HR group was sampled during a period of low daily COVID-19 transmission and its sampling ended before the aforementioned peak. Afterwards, new daily cases stayed above 1,000 until the middle of July when they started to decrease steadily, reaching 219 new cases in the 27th of September (our last sampling date) [62, 63]. When looking into SARS-CoV-2 attack rates in occupational risk groups in Quito, several studies using Real-Time Quantitative Reverse Transcription Polymerase Chain Reaction (RT-qPCR) have reported infection rates of 20.7%, 12.3%, and 15.2%, amongst funeral home workers, police officers, and food delivery riders, respectively [55-57]. No differences in infection rates were seen according to age and sex [55-57]. Other studies, looking at community transmission in other regions of Ecuador have reported variable infection rates ranging from 6.74% to 49% [58-61]. A study by Morales-Jadan et al., using RT-qPCR in four, mostly Andean, provinces of Ecuador (Bolivar, Chimborazo, Tungurahua, and Napo) observed an overall PCR positivity of 26.2% (95%CI, 23.6%-29%); however, when looking into the individual provinces, Tungurahua had the highest infection rate with 65.3% (95%CI, 58.7-71.4%) [58]. This finding is relevant for our study given that Ambato, where our MR group was recruited, is the capital city of Tungurahua; this might also explain the high seropositivity (74.6% [CI 95% 66.4-81.4]) we found in this group. Another study by Rodriguez-Paredes et al., done in the coastal province of Manabí, reported an overall RT-qPCR positivity of 16.13%; however, when looking into individual communities they found infection rates as high as 35% in El Carmen and 33% in Pedernales [59]. All of the aforementioned studies were done from April to September, 2020, 9-12 months prior to our sampling period; however, these studies help to showcase that infection rates in Ecuador were high amongst occupational groups as well as communities in the Andean, Coastal and Amazonian regions, resulting in extensive community transmission since 2020. Therefore, our high seropositivity results, in all occupational exposure groups, respond to this phenomenon of high SARS-CoV-2 attack rates, high community transmission and poor management by the government at the beginning of the epidemic in Ecuador.”*

**Comment 11:** In line 85-86 it states that the recruitment in the HR group started in January 2021 and that the LR and MR groups in July 2021, but how long did it the sampling continue for the different groups? What was the range of the sampling time? If the LR and MR groups were sampled 6 months later, could that explain why there were more seropositive samples among LR than HR? Update: The recruitment dates are given in Table 1. The HR group was sampled between January and February 2021 and the MR group was sampled between 21 July and 27 September. This is unfortunate. How did the pandemic develop between January and September 2021 in Ecuador?

*Between January and February, 2021 (recruitment period of the HR group), a total of 105,724 new COVID-19 cases were reported in Ecuador. More information regarding the development of the pandemic during our sampling period has been added to the discussion section starting at line 279 and now reads: “In Ecuador, surveillance of SARS-CoV-2 infections was carried out by the Public Ministry of Health and reported in official websites and bulletins; from these, during the sampling period of our study (January to September, 2021), a peak in daily COVID-19 confirmed cases (2,085 new cases) was reported in the 27th of April, representing a 2.5-fold increase when compared to the 834 new cases reported in the 13th of January (the beginning of our sampling) [62, 63]. Therefore, our HR group was sampled during a period of low daily COVID-19 transmission and its sampling ended before the aforementioned peak. Afterwards, new daily cases stayed above 1,000 until the middle of July when they started to decrease steadily, reaching 219 new cases in the 27th of September (our last sampling date) [62, 63].”*

**Comment 12:** Line 118-119: the numbers of participants in each group add up to 882, not 873.

*Thank you for pointing this out, it has been corrected, starting at line 173 and now reads: “Of 882 participants included in the analysis, 422, 127, and 333 were in HR, MR, and LR groups, respectively.”*

**Comment 13:** The LR group was much younger than the other two groups. Did you try to age-match the participants? Younger individuals could be less worried of getting COVID-19 and behave differently than older individuals.

*We did not try to age-match the participants because we wanted to analyze the prevalence in three different occupational exposure risk groups. As one of the groups was a university, we expected to have a younger population. We understand that this is a limitation in our study which is why we have analyzed the determinants within each group rather than directly comparing HR vs MR vs LR, as this will lead to biased results.*

**Comment 14:** According to Table 1, there was also a sex difference between the groups with HR and MR being 66.4% and 61.4%, respectively, but the LR groups was only 37.2% women. The individuals in each group should ideally have been more similar in age, samples collected in the same region and at the same time point to optimize the comparison between the groups.

*Thank you for your comment, we understand that these are limitations to our study. We have analyzed the determinants within each group rather than directly comparing HR vs MR vs LR, to avoid bias. Additionally, recruitment at the same time point was not feasible given the government-imposed mobility restrictions (curfews) which prevented us from accessing the LR and MR populations sooner.*

**Comment 15:** Was the access to PCR-testing similar in the different regions? Or was it easier access to testing in the HR group?

*PCR testing was more accessible to the HR group given that this group worked in a public hospital and were regarded by the government as a high priority group that was constantly tested. Moreover, this group was also given priority with vaccination and started receiving vaccines by the end of February and beginning of March, 2021; which is why this was the first group to be recruited as our study required vaccine-naïve participants.*

**Comment 16:** Table 1: in the HR risk, 38.4% reported to have been positive. This is very close the number of positive samples in this group (39.9%). I think it is surprising that there has not been more undetected disease in this group. Usually, seropositive numbers are higher than reported cases. Is it because this group was tested very frequently?

*Yes, we believe that this is due to the frequency of testing in this group given that they were essential workers in a public hospital and were categorized as a priority group by the government.*

**Comment 17:** Table 1: The MR group had a shorter time from COVID-19 diagnosis than the other two groups (95% CI did not overlap for the HR and MR groups). Could this have influenced the seropositivity with waning of antibodies?

*Thank you for bringing this up. We would like to point out that the values in parenthesis are not the 95% CI, as shown in the variable column (first column) they represent the first and third quartiles of the Median, expressed in days. Additionally, since healthcare workers were amongst the first to be exposed to SARS-CoV-2, it was expected for them to have had a longer time from COVID-19 diagnosis.*

**Comment 18:** Table 1- age: I don’t understand the Age category. Is it the SD or the range of age that is shown? Is age given only for positive cases? I think it should be given for all participants in the group with the mean/median and range. And possible between infected/non-infected if there was an age difference between these groups.

*The age is expressed as mean with the SD in parenthesis, in the next column age is expressed as a range. The values shown are for all of the participants in the group. Regarding the ages of the infected/non-infected in each group, there are no major differences between them: for the HR group, the negatives had a mean age of 40.3 (SD=9.9) with a range of 20-65 years and the positives had a mean age of 40.9 (SD=9.8) with a range of 23-69 years; for the MR group, the negatives had a mean age of 39.7 (SD=15.2) with a range of 18-80 years and the positives had a mean age of 40.2 (SD=15.8) with a range of 18-85 years; finally, for the LR group, the negatives had a mean age of 25.4 (SD=8.0) with a range of 18-55 years and the positives had a mean age of 25.6 (SD=7.9) with a range of 18-52 years.*

**Comment 19:** Results. Line 144. It seems that the MR group was significantly different from the other two groups based on the 95% CI.

*Yes, we found a higher seropositivity in the MR group when compared to the others. This can be due to the differences in sample size, sampling period, and/or occupational exposure risk. The participants from the MR group are merchants/farmers that couldn’t self-isolate due to the nature of their work that involves microtransactions in cattle fairs or markets.*

**Comment 20:** Line 151, a questionnaire is mentioned with 20 symptoms. Is this questionnaire added supplementary data? There are only 5 symptoms included in table 2. How very these 5 chosen from the 20 in the questionnaire?

*Thank you for raising this point, we have included the questionnaire as a supplementary file. As explained in the methodology section (lines 87-91), the questionnaire is the ISARIC-WHO Global COVID-19 Clinical Platform Questionnaire (subsections 1b, 1f, and 3a). The 5 symptoms were chosen based on the 5 most frequently reported symptoms in COVID-19 patients^1,2^. We have explained this in the methodology section starting at line 137 and now reads: “Additionally, we selected five symptoms (cough, fever, myalgia, headache, loss of taste and/or smell) to be analysed separately against seropositivity; these symptoms were chosen based on their commonality of presentation in COVID-19 patients [53,54]”*

1. *Stokes EK, Zambrano LD, Anderson KN, Marder EP, Raz KM, El Burai Felix S, Tie Y, Fullerton KE. Coronavirus Disease 2019 Case Surveillance - United States, January 22-May 30, 2020. MMWR Morb Mortal Wkly Rep. 2020 Jun 19;69(24):759-765. doi: 10.15585/mmwr.mm6924e2. PMID: 32555134; PMCID: PMC7302472.*
2. *54. Tong JY, Wong A, Zhu D, Fastenberg JH, Tham T. The Prevalence of Olfactory and Gustatory Dysfunction in COVID-19 Patients: A Systematic Review and Meta-analysis. Otolaryngol Head Neck Surg. 2020 Jul;163(1):3-11. doi: 10.1177/0194599820926473. Epub 2020 May 5. PMID: 32369429.*

**Comment 21:** Lines 160-165 mentions a subgroup analysis of the HR group between those with direct patient contact versus minimal patient contact. How many participants were included (overall and in the two groups)? This finding is also mentioned in the conclusion, lines 277-278 and more information about these sub-analyses should be included.

*Thank you for your useful suggestion, we believe that this will greatly improve the clarity. We have added a paragraph describing this subgroup analysis in greater detail starting at line 226 and now reads: “In a sub-group analysis, we stratified HR participants into hospital personnel with direct patient contact (i.e., doctors, residents, medical students, nurses, nursing assistants, pharmacists, laboratory personnel, and nutritionists), and those personnel with minimal patient contact (i.e., administrative staff, kitchen, security guards, and maintenance). The first group consisted of 344 participants (67.4% women) with a mean age of 40.5 years (SD=9.8), while the second group consisted of 78 participants (61.5% women) with a mean age of 40.9 years (SD=10.3). We observed a greater risk of seropositivity among HR personnel with direct compared to minimal contact (OR 3.30, 95%CI 1.77-5.95, p<0.001).”*

**Comment 22:** Table 3 – was age a continuous variable? I don’t understand the entry.

*Yes, age was a continuous variable and was introduced in the multivariable logistic regression analysis.*

**Comment 23:** Table 2 and 3: There is a line dividing the myalgia row that is not in the other symptom categories. I think this line should be removed.

*We have removed the line.*

**Comment 24:** Discussion. First paragraph: are there any other seroprevalence studies from Ecuador?

*Yes, we discuss the Ecuadorian seropositivity studies starting at line 338, it reads: “Previous seropositivity studies done in Ecuador include several studies from the coastal region of Ecuador, and largely from a single community, Atahualpa, in Santa Elena Province [12–17,19,41], and only one study done in the Andean region on the country in the city of Cuenca [18]. In these studies, the estimated seropositivity ranged from 11.7% (95%CI, 10.0-13.6) to 63.2% (95%CI, 57.4-68.6) [12–19,41]. The pre-vaccination seropositivity observed here, despite sampling having done approximately 1 year after these other studies, remained within this seropositivity range; further, none of these previous studies evaluated potential occupational risks [12–17,19,41].”*

**Comment 25:** Line 185: The 44.6% estimate (overall?) is not mentioned in results.

*Thank you, we have corrected this to reflect the seropositivity of each group, starting at line 187 and now reads: “we found that seropositivity was 39.5% (CI 95% 34.9-44.4) for the HR group, 74.6% (CI 95% 66.4-81.4) for the MR group, and 39.0% (CI 95% 34.0-44.4) for the LR group. “*

**Comment 26:** Reference 38 in line 203, from Colombia, measured seroprevalence using antibodies against the nucleocapsid and not spike/RBD. I found it strange that seropositivity was only 35% after completion of vaccination as most vaccines at that time were based on spike/RBD. I would rephrase this sentence.

*Thank you for pointing this out, we have corrected it starting at line 353 and now reads: “In the Andean region, only two studies have reported seropositivity for HCWs: a study of 1,021 Colombian HCWs reported post-vaccination seropositivity of 35% (95% CI, 31.7–38.6) based on detection of IgG to the SARS-CoV-2 nucleocapsid antigen [38]; and a study 783 Bolivian hospital staff (n=783) before vaccination reported a seropositivity of 43.4% (95%CI, 38.8–48.0) [36], similar to that reported for the HR group of HCWs in our study. Neither of these studies evaluated the effects on seropositivity of direct patient contact (i.e., higher risk of SARS-CoV-2 exposure) [36,38] – here, we observed a 3-fold greater risk of seropositivity among HCWs with direct patient contact compared to those with minimal contact.”*

**Comment 27:** Lines 221-231: about the restrictions in different districts of Ecuador. What were the differences in restrictions between the regions included here?

*One of the regions included in our study is Ambato, its restrictions are described in the paragraph you’re referencing, starting at line 401 and reads: “In Ecuador, each district was responsible for the formulation and implementation biosafety guidelines in their respective populations during the pandemic, such that implementation varied between districts; in Ambato, strict measures were implemented to reduce transmission including fines (equivalent to 10% of the minimum monthly salary) for not wearing a mask, and prohibitions on the of selling of goods in the streets (a common practice in Ecuador and in Ambato) [46]. The latter would have represented an important loss of income for a significant proportion of the population engaged in informal commerce. Temporary migrations of informal merchants to other districts with less severe restrictions or less effective enforcement could have led to a higher risk of SARS-CoV-2 infection” In contrast, in the other region (Quito), given that it’s the capital city of Ecuador, stronger restrictions were in place by the government including mandatory curfew, online work and online education during our sampling periods.*

**Comment 28:** I think the authors could have speculated further on why the seropositivity of the MR group was so high, such as different sampling periods etc.

*Thank you for your suggestion, we have speculated further in the discussion section starting at lines 292, that now reads: “Other studies, looking at community transmission in other regions of Ecuador have reported variable infection rates ranging from 6.74% to 49% [58-61]. A study by Morales-Jadan et al., using RT-qPCR in four, mostly Andean, provinces of Ecuador (Bolivar, Chimborazo, Tungurahua, and Napo) observed an overall PCR positivity of 26.2% (95%CI, 23.6%-29%); however, when looking into the individual provinces, Tungurahua had the highest infection rate with 65.3% (95%CI, 58.7-71.4%) [58]. This finding is relevant for our study given that Ambato, where our MR group was recruited, is the capital city of Tungurahua; this might also explain the high seropositivity (74.6% [CI 95% 66.4-81.4]) we found in this group”; and starting at line 412 that now reads: On the other hand, the higher seropositivity in the MR group in comparison to our two other groups might also be due to the sampling period (i.e., it was the last one to be recruited), although there was some overlap with the LR group [1,33].*

**Comment 29:** Line 143: levels of IgG in relation to loss of taste and smell. These levels are not mentioned or shown in results only positive/negative results.

*Thank you for pointing out this mistake, we have removed the word levels and the sentence, starting at line 479, now reads: “Here, we observed that a history of loss of taste and/or smell was associated with greater seropositivity of IgG antibodies, irrespective of occupational risk group.”*

**Comment 30:** Lines 251-254: I don’t understand what the authors want to say here. The LR group had several symptoms associated with seropositivity, but this was the youngest group. Less severe infections have been more common in younger individuals.

*Thank you for your comment. We would like to point out that we have not discussed infection severity as we did not measure it in our study. In the section you’re referring to, we are discussing that the association between seropositivity and symptomatology can be influenced by sociodemographic factors and rates of asymptomatic or unreported infections. We also cited the EPICOVID study where they found a positive correlation between seropositivity, the symptoms of cough, myalgia, chills and dyspnea, and chronic non-communicable diseases (NCD), which might explain why our HR group (composed of older individuals with more likelihood of having an NCD) have a significant association of seropositivity and myalgias in the multivariate analysis; whereas, the LR composed of younger individuals, have fever as a significant association factor.*

**Comment 31:** The groups with the highest seroprevalence and less positive test, was the MR group (suggesting higher asymptomatic infections. But asymptomatic infections also could give lower levels of antibodies and less seroconversion). Could it be that the MR group was too small to detect associations?

*Thank you for your insightful comment. You raise a very interesting point; however, we don’t believe that it suggests higher asymptomatic infections because the participants in the MR group were not tested frequently. We have failed to make this clear and we appreciate you bringing it up. In our study, only 10 confirmed a positive PCR test, this doesn’t mean that the remaining 117 tested negative. We have included the frequencies of positive, negative and no test in table 1 for each group to improve clarity. In the MR group, 10 participants self-reported having a positive PCR result before our sampling, 6 had a negative PCR result before our sampling, and 111 reported never having a PCR test.*

**Reviewer #2**

**Comment 32:** Materials and Methods: The study is well-done but suffers lack of information on immune responses after immunization. Since immune responses were not evaluated after vaccination and boost, the following sentences should be revised: “analyse changes in antibody levels over a period one year following vaccination with several COVID-19 vaccines”.

*Thank you for your comment. We would like to point out that, as explained in the methodology, starting at line 102, this manuscript represents the baseline analysis of a cohort study that will indeed analyze changes in antibody levels over a period one year following vaccination with several COVID-19 vaccines. However, this being a cross-sectional analysis of the participants before vaccination, we don’t provide that analysis in the current manuscript.*

**Comment 33:** Please, in line 78, clarify “ICUs” acronym.

*The acronym has been clarified in line 122 and now reads: “intensive care units (ICUs),”*

**Comment 34:** Please, in line 107, substitute the “Analyses were conducted on each of three samples” by “Analyses were conducted on each of the samples from three diferent groups”.

*Thank you for your suggestion, we made the replacement.*

**Comment 35:** line 151-154: Whats the difference between symptoms described in your questionnaire and the individual COVID-19 symptomatology? Please, clarify.

*We picked 5 symptoms, from the questionnaire, based on the 5 most frequently reported symptoms in COVID-19 patients^1,2^. We have made this clearer starting at line 137 and now reads:” Additionally, we selected five symptoms (cough, fever, myalgia, headache, loss of taste and/or smell) to be analyzed separately against seropositivity; these symptoms were chosen based on their commonality of presentation in COVID-19 patients [53,54].”*

3. *Stokes EK, Zambrano LD, Anderson KN, Marder EP, Raz KM, El Burai Felix S, Tie Y, Fullerton KE. Coronavirus Disease 2019 Case Surveillance - United States, January 22-May 30, 2020. MMWR Morb Mortal Wkly Rep. 2020 Jun 19;69(24):759-765. doi: 10.15585/mmwr.mm6924e2. PMID: 32555134; PMCID: PMC7302472.*
4. *54. Tong JY, Wong A, Zhu D, Fastenberg JH, Tham T. The Prevalence of Olfactory and Gustatory Dysfunction in COVID-19 Patients: A Systematic Review and Meta-analysis. Otolaryngol Head Neck Surg. 2020 Jul;163(1):3-11. doi: 10.1177/0194599820926473. Epub 2020 May 5. PMID: 32369429.*

**Comment 36:** Please, to all tables, show the significant difference found with a sign and describe it the table footer.

*Thank you for the suggestion, we have included the symbol (*¶) *to denote significance in tables 2 and 3.*

**Comment 37:** Please, write the questionnaire used in the study during recruitment period and post it as supplementary material.

*Thank you for your suggestion, the questionnaire has been added to the supplementary files.*

**Reviewer #3**

**Comment 38:** The period of analysis is from January to September 2021. Is is possible to show the values for each risk group and for the whole month for each month of the study, or at least bi-monthly? My impression is that community transmission was happening from the beginning of the pandemic so perhaps as early as January 2021, the authors will find the same high seropositivity values.

*Thank you for your question, we agree that it would have been interesting to have had a monthly or bi-monthly follow-up; however, the period between January to September 2021 represents the recruitment and sampling period for all of the groups and only one sample was recollected from each participant during this period, before receiving their COVID-19 vaccine.*

**Comment 39:** These results endorsed that SARS-CoV-2 community transmission was happening in Ecuador since early stages of the pandemic. This is why no matter the risk group considered, the values are really high. I am missing important references and a wider discussion of this topic in this manuscript. There are several articles done in Ecuador during the first semester of COVID-19 pandemic that even using RT-PCR testing, a non cummulative approach like serology to address prevalence of infection, were able to detect really high attack rates. Interestingly, three of this manuscript refers to risk occupational groups like police, food delivery riders or funeral home workers (See the DOI for this papers: DOI: 10.3389/fpubh.2022.1012434; DOI: 10.3389/fmed.2021.735821; DOI: 10.1016/j.scitotenv.2021.145225). This papers should be discussed in the context of this manuscript as clearly endorse the same findings that the authors get on this study: a high prevalence of SARS-CoV-2 infection on occupational risk groups.

*Thank you for your comment and for suggesting the articles referenced above. We read the suggested articles with great interest and have now added them to the references and commented on our results in their context starting at line 288 and now reads: “When looking into SARS-CoV-2 attack rates in occupational risk groups in Quito, several studies using Real-Time Quantitative Reverse Transcription Polymerase Chain Reaction (RT-qPCR) have reported infection rates of 20.7%, 12.3%, and 15.2%, amongst funeral home workers, police officers, and food delivery riders, respectively [55-57]. No differences in infection rates were seen according to age and sex [55-57]. Other studies, looking at community transmission in other regions of Ecuador have reported variable infection rates ranging from 6.74% to 49% [58-61]. A study by Morales-Jadan et al., using RT-qPCR in four, mostly Andean, provinces of Ecuador (Bolivar, Chimborazo, Tungurahua, and Napo) observed an overall PCR positivity of 26.2% (95%CI, 23.6%-29%); however, when looking into the individual provinces, Tungurahua had the highest infection rate with 65.3% (95%CI, 58.7-71.4%) [58]. This finding is relevant for our study given that Ambato, where our MR group was recruited, is the capital city of Tungurahua; this might also explain the high seropositivity (74.6% [CI 95% 66.4-81.4]) we found in this group. Another study by Rodriguez-Paredes et al., done in the coastal province of Manabí, reported an overall RT-qPCR positivity of 16.13%; however, when looking into individual communities they found infection rates as high as 35% in El Carmen and 33% in Pedernales [59]. All of the aforementioned studies were done from April to September, 2020, 9-12 months prior to our sampling period; however, these studies help to showcase that infection rates in Ecuador were high amongst occupational groups as well as communities in the Andean, Coastal and Amazonian regions, resulting in extensive community transmission since 2020. Therefore, our high seropositivity results, in all occupational exposure groups, respond to this phenomenon of high SARS-CoV-2 attack rates, high community transmission and poor management by the government at the beginning of the epidemic in Ecuador.” We agree that providing a broader view of SARS-CoV-2 infection in Ecuador and we think that this suggestion has improved the scientific message of the paper.*

**Comment 40:** Also, few more manuscripts also endorse extended community transmission in general population even in rural and remote locations in Ecuador. These studied should be also discussed in the context of this manuscript as they endorse the findings of the authors: even in low-risk group the prevalence is really high. This is expected if those results are put on the context of the findings of these mentioned articles that show extensive community transmission all over Ecuador (See the DOI to revise and cite: DOI: 10.3389/fmed.2023.1001679; DOI: 10.4269/ajtmh.21-0582; DOI: 10.2471/BLT.20.283028; DOI: 10.22605/RRH7643).

*Thank you for your comment and for suggesting the articles referenced above. We read the suggested articles with great interest and have now added them to the references and commented on our results in their context starting at line 288 and now reads: “When looking into SARS-CoV-2 attack rates in occupational risk groups in Quito, several studies using Real-Time Quantitative Reverse Transcription Polymerase Chain Reaction (RT-qPCR) have reported infection rates of 20.7%, 12.3%, and 15.2%, amongst funeral home workers, police officers, and food delivery riders, respectively [55-57]. No differences in infection rates were seen according to age and sex [55-57]. Other studies, looking at community transmission in other regions of Ecuador have reported variable infection rates ranging from 6.74% to 49% [58-61]. A study by Morales-Jadan et al., using RT-qPCR in four, mostly Andean, provinces of Ecuador (Bolivar, Chimborazo, Tungurahua, and Napo) observed an overall PCR positivity of 26.2% (95%CI, 23.6%-29%); however, when looking into the individual provinces, Tungurahua had the highest infection rate with 65.3% (95%CI, 58.7-71.4%) [58]. This finding is relevant for our study given that Ambato, where our MR group was recruited, is the capital city of Tungurahua; this might also explain the high seropositivity (74.6% [CI 95% 66.4-81.4]) we found in this group. Another study by Rodriguez-Paredes et al., done in the coastal province of Manabí, reported an overall RT-qPCR positivity of 16.13%; however, when looking into individual communities they found infection rates as high as 35% in El Carmen and 33% in Pedernales [59]. All of the aforementioned studies were done from April to September, 2020, 9-12 months prior to our sampling period; however, these studies help to showcase that infection rates in Ecuador were high amongst occupational groups as well as communities in the Andean, Coastal and Amazonian regions, resulting in extensive community transmission since 2020. Therefore, our high seropositivity results, in all occupational exposure groups, respond to this phenomenon of high SARS-CoV-2 attack rates, high community transmission and poor management by the government at the beginning of the epidemic in Ecuador.” We agree that providing a broader view of SARS-CoV-2 infection in Ecuador and we think that this suggestion has improved the scientific message of the paper.*

**Comment 41:** The authors should provide more details about the clinical performance of the serological test used. It is important to know the sensitivity of this test as this would impact the true values of seropositivity. For instance, the authors cite papers done in communities from Santa Elena and Esmeraldas, where rapid antigen test were used. That means a lost of sensitivity compared to a good ELISA test and probably an underestimation of prevalence of easily 20% or more. I would expect that the ELISA used in this study has a good sensitivity but no matter that the authors should address the estimated true values considering the sensitivity (and also specificity) of the text used.

*Thank you for the suggestion, we have added these values on the methodology section starting at line 152 that now reads: this test had a sensitivity, specificity, positive predictive value, and negative predictive value of 93.6%, 100%, 100%, and 95.4%, respectively [32].*

**Comment 42:** Those results are quite worrying and means that COVID-19 pandemic was really out of control in Ecuador. I am missing an strong discussion about this, contextualizing this results with the mortality in Ecuador associated to COVID-19 (there are also several papers addressing this topic in Ecuador that the authors should consider to cite). Moreover, the scenario described for Ecuador was happening in other countries in Europe or Asia much more later and basically after omicron wave. It is important to discuss this topic as that means that COVID-19 pandemic would have been poorly managed in Ecuador.

*Thank you for your comment, we have referenced the suggested articles and discuss the importance of the high attack rates, community transmission and poor governmental management. We believe that these points have improved the message of our article. The paragraph starts at line 277 and now reads: “It's important to interpret our data in the context of community transmission and SARS-CoV-2 attack rates in order to understand why we obtained high seropositivity amongst all groups, and in particular amongst the MR group. In Ecuador, surveillance of SARS-CoV-2 infections was carried out by the Public Ministry of Health and reported in official websites and bulletins; from these, during the sampling period of our study (January to September, 2021), a peak in daily COVID-19 confirmed cases (2,085 new cases) was reported in the 27th of April, representing a 2.5-fold increase when compared to the 834 new cases reported in the 13th of January (the beginning of our sampling) [62, 63]. Therefore, our HR group was sampled during a period of low daily COVID-19 transmission and its sampling ended before the aforementioned peak. Afterwards, new daily cases stayed above 1,000 until the middle of July when they started to decrease steadily, reaching 219 new cases in the 27th of September (our last sampling date) [62, 63]. When looking into SARS-CoV-2 attack rates in occupational risk groups in Quito, several studies using Real-Time Quantitative Reverse Transcription Polymerase Chain Reaction (RT-qPCR) have reported infection rates of 20.7%, 12.3%, and 15.2%, amongst funeral home workers, police officers, and food delivery riders, respectively [55-57]. No differences in infection rates were seen according to age and sex [55-57]. Other studies, looking at community transmission in other regions of Ecuador have reported variable infection rates ranging from 6.74% to 49% [58-61]. A study by Morales-Jadan et al., using RT-qPCR in four, mostly Andean, provinces of Ecuador (Bolivar, Chimborazo, Tungurahua, and Napo) observed an overall PCR positivity of 26.2% (95%CI, 23.6%-29%); however, when looking into the individual provinces, Tungurahua had the highest infection rate with 65.3% (95%CI, 58.7-71.4%) [58]. This finding is relevant for our study given that Ambato, where our MR group was recruited, is the capital city of Tungurahua; this might also explain the high seropositivity (74.6% [CI 95% 66.4-81.4]) we found in this group. Another study by Rodriguez-Paredes et al., done in the coastal province of Manabí, reported an overall RT-qPCR positivity of 16.13%; however, when looking into individual communities they found infection rates as high as 35% in El Carmen and 33% in Pedernales [59]. All of the aforementioned studies were done from April to September, 2020, 9-12 months prior to our sampling period; however, these studies help to showcase that infection rates in Ecuador were high amongst occupational groups as well as communities in the Andean, Coastal and Amazonian regions, resulting in extensive community transmission since 2020. Therefore, our high seropositivity results, in all occupational exposure groups, respond to this phenomenon of high SARS-CoV-2 attack rates, high community transmission and poor management by the government at the beginning of the epidemic in Ecuador.”*
