# Supplementary material for: COVID-19 IgG seropositivity and its determinants in occupational groups of varying infection risks in two Andean cities of Ecuador before mass vaccination
Source: PLoS One. 2024 Aug 29;19(8):e0309466. doi: 10.1371/journal.pone.0309466 (PMC11361580; doi:10.1371/journal.pone.0309466)
Supplement: Attachment — Submitted filename: Response to Reviewers_2.docx [file pone.0309466.s003.docx]

“COVID-19 IgG Seropositivity and its determinants in occupational groups of varying infection risks in two Andean cities of Ecuador before mass vaccination”

Response to Reviewers

Dear Editor,

We would like to thank you for your consideration for publication of our manuscript in PLOS ONE. We would also like to thank you for the opportunity to reply to the reviewers’ comments, requests, and constructive criticisms.

We have addressed them all bellow point-by-point and have highlighted the changes in the manuscript’s text. We have also checked the accuracy of all the included references. We believe that our article has now fulfilled all requirements to be considered for publication in PLOS ONE.

Dear Reviewers,

Thank you for your insightful comments and constructive suggestions. Below, please find a point-by-point response addressing your remarks.

**Reviewer #1**

**Comment 1:** I am satisfied with the authors’ answers to my questions/comments and the updated manuscript. I support that this manuscript now should be accepted.

*Thank you for your recommendation, your suggestions have certainly improved the clarity of our manuscript.*

**Reviewer #3**

**Comment 1:** The authors have improved the manuscript with a better contextualization of their results. However, there is one of my comments that I believe it was not fully understood. I ask the authors to provide the values for seropositivity for each month of the study, or bi-monthly at least. This is possible to do no matter if only one sample was collected for each individual. My idea with this comment is to see if there was any trend in seropositivity of if those high values found by the authors were already happening as early as January 2021, what would mean that COVID-19 was spreading massively along 2020 in the Ecuadorian population. I believe this analysis will make the manuscript even more interesting. It is up to the authors to include this final recommendation in the final version.

*Thank you for your comment. To improve clarity, we have provided the recruitment dates for each of the three risk groups in the text of Methods. Recruitment of the 3 groups was sequential starting with the HR group and was done between January and September. Seropositivity by month of recruitment is shown in the graph below. As we can see the data are insufficient to allow evaluation of a trend in the transmission of SARS-CoV-2 in these baseline data.*
